# Hæmaturia as a Symptom of Diseases of the Genito-Urinary Organs

**Published:** 1884-08

**Authors:** A. R. Davidson

**Affiliations:** Professor of Medical Chemistry, Toxicology and Diseases of the Skin, Niagara University


### BUFFALO

### Medical and Surgical Journal.

Vol. XXIV. AUGUST, 1884. No. 1.

### Original Communications.

### Hæmaturia as a Symptom of Diseases of the Genito-Urinary Organs.*

By A. R. Davidson, M. D.,

Professor of Medical Chemistry, Toxicology and Diseases of the Skin, Niagara University.

It is by no means a rare thing to find patients voiding bloody urine. If the blood is in small quantity, and especially if it comes from the kidney, the patient may not recognize it, and even a physician may easily overlook it; but when in sufficient quantity to give the urine its distinctive red color it never fails to cause great alarm to the patient and an immediate appeal for medical aid.

Although hæmaturia cannot be regarded as a distinctive disease, still its importance as a symptom, oftentimes, of the most formidable diseases of the urinary tract, and the greater importance of being able to recognize its origin and import, leads me to hope that a consideration of the subject may not be without interest to the members of the association. Those most familiar with urinalysis will be very willing to acknowledge that it is not always an easy thing to determine from what

*Read before the Buffalo Medical and Surgical Association.

point in that long and complicated organic apparatus, which commences in the malpighian body and terminates at the external meatus, the blood in question originates.

The term hæmaturia is commonly used to designate any condition in which blood is present in the urine. The name, however, should be restricted to those cases in which blood is present in its entirety, that is, including the red corpuscles. In many cases the absence of these is demonstrable by the microscope, when by the spectroscope and other means we show clearly the presence of the coloring matters of the blood, either in the form of hæmoglobin or hæmatin. The presence of the first is designated by by the term hæmoglobinuria, and the second by hæmatinuria. The distinction is important in regard to pathological significance. In hæmaturia a true hemorrhage has occurred in some part of the urinary tract. In hæmoglobinuria, or hæmatinuria, the inference from the absence of blood corpuscles is that a dissolution of them has taken place within the blood vessels and the coloring matter excreted by the kidney. This condition occurs especially with those diseases accompanied by a so-called dissolution of the blood, as scurvy, malignant intermittent fever, putrid typhus, hæmophilia, etc., and it may be observed after the transfusion of a considerable quantity of animal blood into the human organism. But this evening we will consider only true hæmaturia, or, rather, I will endeavor to recall to your minds those affections of the urinary apparatus with which hemorrhage is a frequent symptom, and show you how far the intelligent study of this symptom alone, may lead us in our diagnosis of the disease.

The blood may come from any part of the urinary apparatus, and we may conveniently divide it, according to its character, into two classes—profuse hemorrhage, from rupture of the larger vessels, and parenchymatous hemorrhage.

Profuse hemorrhage usually comes from the pelvis of the kidney, the urethra, or the bladder, rarely from the kidneys themselves. The urine is colored red, or dark reddish yellow, and upon standing for some hours may deposit the entire blood, leaving the urine of a normal color. If the urine is ammoniacal there will be a solution of the blood-coloring matter. The reaction is usually neutral, or alkaline, and the specific gravity varying. Albumen is always present from the serum of the blood.

Parenchymatous hemorrhage usually comes from the kidney, and may come from the bladder and the entire urinary tract. The urine is red-brown, often coffee-colored. After standing for some time it deposits a slight sediment, but the supernatant liquid retains its high color, because the blood-coloring matters are partly in solution. The reaction is usually acid and the specific gravity lowered.

Profuse hemorrhage may be an accompaniment of the following conditions: In the urethra—polypoid growths, or vascular tumors, diseases of the prostate. In the bladder—varix, or hemorrhoids, calculus, and catarrhal ulceration of the neck, villous tumors, or papilloma. In the pelvis of the kidney—from cancer and renal calculi.

It, of course, also may accompany traumatic injuries, but it is rare that we look to the urine for a diagnosis in these cases, so we omit mention of them.

By the term "profuse hemorrhage" I do not mean you to understand that in all these cases mentioned there is necessarily a large amount of blood in the urine, but rather that the characteristics of the blood present point clearly to a bleeding from a vessel in contra-distinction to the oozing which takes place in parenchymatous hemorrhage. We are not always able to make this distinction hold, as, for instance, in bleeding from the prostate the blood may be regurgitated back into the bladder, and when voided present all the appearances of parenchymatous hemorrhage. The marked difference in the appearance of the urine, however, as before stated, caused the older practitioners to make the distinction between kidney and bladder hemorrhages simply upon that, and the exceptions are so few that the division is a convenient one even now.

Having, then, a specimen of urine which falls into the class of "profuse hemorrhage," how are we to make our differential diagnosis? If the disease is anterior to the bladder it may usually be demonstrated by causing the patient to void his urine into two glasses. The first contains nearly all the blood, if its origin is in the urethra. The urine will contain blood corpuscles of normal size and no peculiar features. The sound and digital examination of the prostate by the rectum will settle our diagnosis.

If the blood proceeds from the bladder the diagnosis is not so easy. If it arises from bladder hemorrhoids the bleeding may be profuse. An instance occurred in the Hotel Dieu, Paris, in which the bleeding was so abundant as to prove fatal. The autopsy revealed the presence of several varices at the neck of the bladder, upon one of which was a large ulcer, from which the bleeding had evidently proceeded. The bladder was healthy in all other respects. The disease is most common in old age, but is sometimes met with in youth. The diagnosis is difficult and sometimes wholly impossible.

Calculus in the bladder is a common cause of hemorrhage, but rarely in sufficient quantity to require treatment. It usually comes on after some violent exercise. In these cases the well-known symptoms of stone are present, and an examination with the sound removes all doubt as to the cause.

The hæmaturia from catarrhal ulcerations, which occur in the neck of the bladder usually after gonorrhœa, exhibits itself toward the close of micturition, when the sphincter vesicæ begins to contract. The urine will be alkaline, and in the sediment we find, beside blood, pus corpuscles, crystals of ammonia, magnesium, phosphate and bladder epithelium. Villous tumors and papilloma rarely give rise to profuse hemorrhage, unless the cancer has existed for a long time and become ulcerated, or if by any cause there is produced strong muscular contraction of the bladder, the blood vessels may be so violently compressed that the tension in the vessels of the villous tumor may be great enough to rupture them. In this case it is rare that we are able to find necrotic cancer tissue, and we may seek in vain for any indication in the urine to account for the hemorrhage. Villous cancer, however, usually involves the back and under wall of the bladder, so that the thickening, or tumor, may be felt by introducing the finger into the rectum. Papilloma of the bladder, on the contrary, is confined simply to the mucous membrane, and we are unable to find a thickening of the bladder wall or a tumor from rectal investigation. Hoffman states that papilloma frequently passes over into villous cancer. In the commencement of the disease there will be only a parenchymatous hemorrhage, and careful examination will always show branches of villous tissue with very thin epithelial covering.

Severe hemorrhages from the kidneys are very rare. When they do occur, from cancer, or ulcerations produced by renal calculus, the quantity of blood corpuscles present makes it very difficult to find the kidney epithelium, the recognition of which is essential to a diagnosis.

Parenchymatous hemorrhage may come from the kidney and its pelvis, or the bladder, or the entire urinary apparatus, and, as before stated, the urine is of a red-brown or brown-black color, because the blood mixes gradually, drop by drop, with the urine, the corpuscles remaining a long time with a relatively great amount of fluid. By this intimate mixing the urinary constituents have time to exert their destructive influence on the blood corpuscles and convert the red hæmoglobin to the brown methhæmoglobin. Such a hemorrhage we have in the majority of cases of acute or chronic parenchymatous nephritis; regularly with atheromatous degeneration of the kidney vessels, constantly with renal calculi, though no severe pyelitis may be present, and with cancer of the kidney.

Pathognomonic of renal hemorrhage, as parenchymatous hemorrhage, is, we must not forget, the exception with papilloma, as before stated, of the bladder, and with carcinoma villosum it is also present, and a slow bleeding from the prostate, in which the blood is regurgitated into the bladder, may give rise to all the appearances of this form of hemorrhage; and lastly, we may have a parenchymatous hemorrhage from the entire urinary apparatus. For example: If for some time a paralyzed bladder has been only partially emptied by micturition, and suddenly the entire amount is drawn off by a catheter, there arises, necessarily, a hyperæmia, *ex vacuo*, which is the more intense according as the muscles of the bladder have become hypertrophied, rendering complete contraction impossible. The kidney, also, is suddenly relieved from the secretory pressure necessary to overcome the weight of the residual urine in the bladder, and a parenchymatous hemorrhage ensues.

In the differential diagnosis of these hemorrhages microscopic analysis furnishes the greatest aid. If it is an accompaniment of acute or chronic parenchymatous nephritis, it is associated with casts and kidney epithelium, and is only one of a series of indications, the careful study of which will enable us, in most cases, to diagnose the conditions of the kidney in the formidable disease which, under the indefinite collective term of Bright's disease, embraces all the varieties of diseases of the renal parenchyma. Intensely interesting as the study of the indications by which we can acquaint ourselves with the condition of the kidney and form a reasonable prognosis, the limits of this paper forbid our entering into it.

With renal calculi, we find in the sediment, besides blood corpuscles of various sizes and kidney epithelium, jagged crystals of uric acid or calcium oxalate, and usually a more or less severe pyelitis is present. With cancer of the kidney, besides the parenchymatous hemorrhage, we may find carcinomatous tissues in the sediment, usually in the form of small masses, aggregations of cells with thick walls or caudate and long spindle-shaped cells.

Cancer of the bladder is generally a so-called villous cancer, that is, it is made up of compound, branched villi, which are sometimes hollow and composed of fibrous stroma covered with a layer of variously shaped epithelial cells. It is a mistake to suppose that you will find this villous tissue unchanged, and presenting the beautiful forms represented in plates. Such can, however, be observed when, accidentally, a small fragment of the fresh growth has been detached and brought out in the orifice of a catheter, but necrotic, villous tissue may appear in various forms, which are sufficiently characteristic to enable us to diagnosticate such diseases of the bladder with certainty. The urine shows the following characteristics: It is red-brown or blackish in color. Undisturbed, there is present, in addition to the blood, pus corpuscles, which increase with the growth of the villous tumor. The excretion of normal matter is not altered. Albumen and blood-coloring matters are found in considerable quantity, and there is more albumen in the urine than is accounted for by the amount of pus and blood in the sediment. This condition depends upon the increased tension within the supplying vessels of the villous tissue.

One must be careful in such cases not to diagnose a kidney affection unless kidney cylinders are distinctly recognized, as the other indications are not unlike those of true albuminuria; and, indeed, it is easy for those unaccustomed to the appearances of them to mistake the single villous shreds for renal casts. Cancer of the kidney is usually much more difficult to diagnosticate. Sometimes when cancer cells are present in the urine we can determine the existence of the renal disease by negative signs, as all symptoms pointing to a disease of the bladder are wanting. In the early stages of the disease it is often impossible to discriminate between it and calculous pyelitis. In some cases there may be no hemorrhage, and the urine may be, from first to last, healthy—due to the fact that the ureter of the affected side often becomes impervious at an early stage of the disease.

In locating the origin of the blood flow the blood coagula often give valuable information. If they are soft and of the color and consistence of freshly clotted blood, they must be of very recent formation. Clots which have been in the urinary apparatus for some time are harder and assume a dirty yellow color. If from the bladder, they are usually soft and irregular in form, while from the kidney they are apt to be long and rod-shaped. They are seldom present in parenchymatous hemorrhage, but the so-called blood cylinders and the hemorrhagically-tinged kidney epithelium are characteristics almost always present if it proceeds from the kidney. Blood casts consist of coagulated fibrin with blood corpuscles entangled.

There are very exceptional cases in which, even after a careful examination of the fluid from the bladder, we will be unable to entirely satisfy ourselves as to the source of the blood. In such cases we may remove all doubt by collecting the urine as it flows from the ureters. Sir Henry Thompson was, I think, the first to practice and recommend a mode by which this is easily accomplished, as follows: A soft rubber catheter of medium size is passed into the bladder, the patient standing; all of the urine is drawn off and the viscus repeatedly washed out by small injections of warm water. The urine is then permitted to pass, as it will do guttatim, into a test tube or other small vessel, for purposes of examination. The bladder ceases for a time to be a reservoir; it does not expand, but is contracted around the catheter, and the urine percolates from the ureters direct. A specimen can thus be obtained free from all admixture with products of the bladder or urethra, and will often furnish the only data previously wanting to accomplish an exact diagnosis.

